# Social Achievement Goals in Chinese Undergraduates: Associations With Self-Esteem and Symptoms of Social Anxiety and Depression

**DOI:** 10.3389/fpsyg.2022.726679

**Published:** 2022-04-13

**Authors:** Yanhua Zhao

**Affiliations:** Department of Psychology, Institute of Cognition, Brain and Health, Henan University, Kaifeng, China

**Keywords:** social achievement goals, self-esteem, social anxiety symptoms, depression symptoms, undergraduates

## Abstract

The pursuit of relationship goals is critical to the wellbeing of young adults. This study investigated different achievement goals toward social competence as potential predictors of social anxiety and depression symptoms. It proposed that self-esteem may function as a mediator on the pathway from endorsing social achievement goals to undergraduates' concurrent and longitudinal social anxiety and depression symptoms. Social achievement goal theory proposes three types of goals: social mastery goals (striving to improve one's social competence), social performance-approach goals (striving to prove one's social competence and win positive evaluation), and social performance-avoid goals (striving to avoid incompetent social behaviors and negative evaluation). One hundred and eighty-five Chinese undergraduates aged from 18 to 23 (50% female) completed this study across two-time points. Path analyses indicated that social mastery (marginally) and performance-approach goals were positively associated with self-esteem, whereas social performance-avoid goals were negatively associated with self-esteem; self-esteem was negatively associated with the concurrent social anxiety and depression symptoms and the longitudinal depression symptoms. The proposed mediation effects of self-esteem on the links from three types of social achievement goals to the concurrent and longitudinal social anxiety and depression symptoms were significant except on the links from social mastery goals and social performance-approach goals to the subsequent social anxiety symptoms. Self-esteem and the baseline social anxiety and depressive symptoms have a chain mediating effect between social achievement goals and the longitudinal symptoms of social anxiety and depression. These findings suggest that the pursuit of social mastery goals and performance-approach goals in initiating and maintaining social relationships boosts undergraduates' self-worth and reduces their concurrent and longitudinal depression experiences. However, the strivings to hide inadequacy and avoid negative evaluation in social contexts impede one's self-worth and increase concurrent and longitudinal social anxiety and depression symptoms. Implications and limitations are discussed.

## Introduction

Social anxiety and depression are common psychological problems with a high prevalence rate among young adults (Ranøyen et al., 2018; Jefferies and Ungar, 2020). COVID-19 has further exacerbated the epidemic trend of psychological distress (Bueno-Notivol et al., 2021; Hawes et al., 2021). Experiencing excessive affective disturbance undermines youth's ability to thrive in academic and social domains and increases the risk for suicide (Chartrand et al., 2011; Brook and Willoughby, 2015; Ribeiro et al., 2018; Hur et al., 2019). In China, more than 8% of college students in Daqing city have been diagnosed with clinical social anxiety (Luan et al., 2014), and the prevalence rate of clinical depression among college students over the 10 years from 2009 to 2019 was 31.4% (see meta-analysis, Wang et al., 2020). Thus, understanding the developmental psychopathology of social anxiety and depression is particularly meaningful for improving young people's mental health in transition to adulthood.

Some researchers have proposed that individual differences in social goals and self-esteem can help explain why some people are particularly susceptible to affective disorders (Cheng et al., 2015; Li et al., 2015; Shin and Park, 2022). For example, the pursuit of different achievement goals in developing and maintaining social relationships has been connected with undergraduates' social anxiety and depressive symptoms (Kuroda and Sakurai, 2011; Shim and Ryan, 2012). Self-esteem, which signifies the overall sense of self-evaluation people make across various situations (James, 1890), is another solid contributor to social anxiety and depression (Schreiber et al., 2012; Hiller et al., 2017; Kim and Moore, 2019; Gao et al., 2022). Undergraduates, who endorse different achievement goals toward social competence (Ryan and Shim, 2006), are experiencing a critical period of identity exploration and consolidation (Arnett, 2000). Although theories and empirical studies have suggested that self-esteem is an important predictor in the development of social anxiety and depression, and self-esteem can be predicted by different personal achievement goals (Chen et al., 2017; Ferradás et al., 2019), no study has examined whether self-esteem can serve as a mediator between achievement goals and symptoms of social anxiety and depression. In the current study, we aimed to investigate whether the endorsement of different social achievement goals is associated with one's self-esteem and whether self-esteem may serve as a mediator explaining the relation between social achievement goals and undergraduates' concurrent and longitudinal symptoms of social anxiety and depression.

## Social Achievement Goals

Social achievement goal theory is a three-dimensional framework developed by Ryan and Shim (2006) that applies achievement goal theory (Dweck and Leggett, 1988) to the social domain. This framework proposes three types of goals concerning mastery and performance (approach and avoid) orientations toward social competence, which has demonstrated important implications for adolescents' and undergraduates' adjustment (Ryan and Shim, 2006; Horst et al., 2007; Shim et al., 2013a; Liem, 2016; Bardach et al., 2020). *Social mastery goals* focus on striving to develop and improve one's social relations and social skills, and reflect the importance one places on social relationships (e.g., having friends who understand me, having friends who care about me). These goals have been associated with more adaptive outcomes such as positive social relationships, social competence, self-efficacy, perceived popularity, and a stronger sense of belonging (Ryan and Shim, 2006; Horst et al., 2007; Shim and Ryan, 2012; Choi and Park, 2018; Bardach et al., 2020; Chang and Hall, 2022), and with less maladaptive outcomes such as depressive symptoms, social withdrawal, sense of loneliness, and aggressive behaviors (Mouratidis and Sideridis, 2009; Kuroda and Sakurai, 2011; Choi and Park, 2018; Bardach et al., 2020; Kim and Park, 2021).

*Social performance-approach goals* focus on seeking to prove one's social competence and winning positive judgments (e.g., wishing to be seen as having a lot of friends, seeking to be friends with popular people). These goals have shown contingent results in predicting undergraduates' adjustment. They have been found to be beneficial for improving social popularity and for reducing social withdrawal (Shim and Ryan, 2012; Choi and Park, 2018; Kim and Park, 2021) and depressive symptoms (Kuroda and Sakurai, 2003, 2011), but maladaptive in increasing aggressive behaviors and social worry (Ryan and Shim, 2006; Horst et al., 2007; Shim et al., 2013a; Choi and Park, 2018; Bardach et al., 2020). *Social performance-avoidance goals* focus on avoiding performing socially incompetent behaviors (e.g., being socially awkward or “goofing off”) and avoiding negative evaluation from others (e.g., being perceived as foolish or a “loser”). These goals have been associated with more maladaptive outcomes such as interpersonal stress, social worry, social anxious behaviors, depressive symptoms, and loneliness, and with less adaptive outcomes in the areas of social relationships, social efficacy, perceived popularity, and social competence (Ryan and Shim, 2006; Horst et al., 2007; Kuroda and Sakurai, 2011; Shim and Ryan, 2012; Liem, 2016; Choi and Park, 2018). In sum, previous studies indicated that social achievement goals could uniquely contribute to youngsters' social adjustment and social anxiety and depressive symptoms, especially when these goals are combined with other psychological constructs (Ryan and Shim, 2006; Kuroda and Sakurai, 2011; Shim et al., 2013b; Choi and Park, 2018; Kim and Park, 2021).

## Social Achievement Goals and Self-Esteem

Theories and empirical studies suggest that pursuing social achievement goals can influence one's self-esteem. Essentially, either success or failure to attain goals impacts one's self-esteem (Greenberg and Pyszczynski, 1985; Heatherton and Polivy, 1991; Bongers et al., 2009). In Covington's (1984) self-worth theory, the ability is central to the estimation of self-worth, and young people can be motivated to establish and sustain a reputation of competency to protect this. According to sociometer theory, self-esteem is a psychological gauge of one's perceived self-value as a relational partner, and the perception of whether they are socially valued strongly affects self-esteem (Leary and Baumeister, 2000). People endorsing social mastery goals focus on learning new things and personal growth, and even a slight improvement of social abilities and quality of relationships can be seen as successful to them (Ryan and Shim, 2006). Because social ability and higher quality of social relationships are positively associated with self-esteem (Covington, 1984; Denissen et al., 2008; Stinson et al., 2008; Hapsari and Sholichah, 2021), the pursuit of social mastery goals provides many opportunities for enhancing one's self-esteem. Consistent with this reasoning, the literature states that people with social mastery goals tend to report higher self-esteem in academics (Shim et al., 2012) and the social domain (Shim and Ryan, 2012).

With social performance-approach goals and social performance-avoid goals, people concentrate on the appearance of their social selves (Ryan and Shim, 2006). People endorsing social performance-approach goals focus on impressing others, so they judge their success based on others' judgments and standards. Because social performance-approach goals have been positively associated with self-efficacy (Kim and Park, 2021), the perception of self-efficacy may further strengthen one's self-esteem. Individuals who endorse social performance-avoid goals prioritize hiding inadequacy and avoiding a negative reputation. These individuals tend to develop less positive relationships with others and demonstrate the lower social ability and self-efficacy (Ryan and Shim, 2006; Horst et al., 2007; Shim and Ryan, 2012; Kim and Park, 2021), which, in turn, threatens one's self-esteem (Covington, 1984; Denissen et al., 2008; Stinson et al., 2008). People endorsing social performance (both approach and avoid) goals tend to report lower self-esteem (Shim and Ryan, 2012). In a longitudinal study, Shim et al. (2012) found that the initial performance-avoid goals and the increases of performance-approach goals in the academic domain related to more maladaptive change trajectories of self-esteem during the first academic year of college students.

## Role of Self-Esteem

The significant impacts of self-esteem on anxiety and depression symptoms have been documented in previous longitudinal studies and meta-analyses (Orth et al., 2008; Sowislo and Orth, 2013; Rieger et al., 2016). Theories from the cognitive perspective, which propose that the negative beliefs of the self in social situations are risk factors for the development of social anxiety and depression (Beck, 1967; Clark and Wells, 1995), provide insights for the study of the etiology of affective disorders. Youth with low self-esteem from Western and Chinese societies are more likely to exhibit social anxiety (Shim and Ryan, 2012; Cheng et al., 2015; Abdollahi and Abu Talib, 2016; Hiller et al., 2017) and depressive symptoms (Li et al., 2015; Rieger et al., 2016; Gao et al., 2022).

Although self-esteem has been found as a promising predictor of social anxiety and depression symptoms, no study to date has tested whether self-esteem can be a mediation mechanism underlying the effects of social achievement goals on the symptoms of social anxiety and depression. As presented in prior sections, achievement goal orientations have been connected to the initial level and subsequent fluctuation of self-esteem in both academic and social domains (Shim et al., 2012), both social achievement goals and self-esteem have a significant influence on undergraduates' social anxiety and depressive symptoms; thus, it is possible that social achievement goals affect social anxiety and depression symptoms through their impact on self-esteem. We hypothesized that self-esteem serves as a mediator on the pathway from social achievement goals to social anxiety and depression symptoms.

## The Present Study

The present study aims to investigate the relationship between social achievement goals, self-esteem, and undergraduates' social anxiety and depression symptoms and to examine whether self-esteem may function as a mediation mechanism on the pathway from social achievement goals to social anxiety and depression symptoms. Given the lack of evidence regarding the social achievement goals on long-term psychopathology symptoms, both concurrent and longitudinal symptoms of social anxiety and depression will be examined.

**Figure 1:**
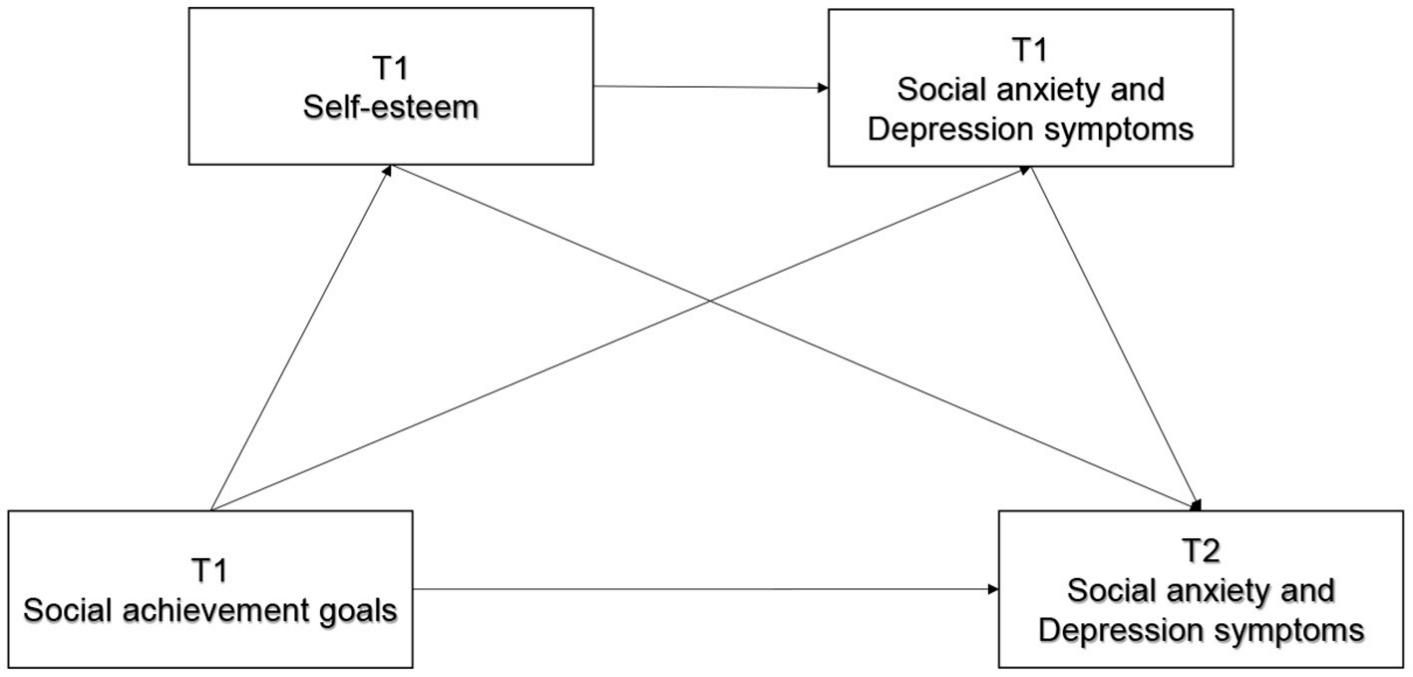
The proposed path model.

Specifically, we examined (1) whether social achievement goals and self-esteem are associated with the self-reported social anxiety and depression symptoms at the baseline level and at a 10-month follow up; (2) whether self-esteem is associated with social achievement goals and constitutes a mediation mechanism underlying the effects of social achievement goals on the concurrent and subsequent symptoms of social anxiety and depression; (3) whether there is a chain mediating effect of self-esteem and the baseline social anxiety and depressive symptoms between three types of social achievement goals and the subsequent symptoms of social anxiety and depression, using a university students sample from mainland China. The proposed model is presented in Figure 1.

Additionally, a recent study showed that women reported higher scores of social anxiety symptoms (Guo, 2021). Another meta-analysis showed that the prevalence of depression among Chinese college students was higher than that of women in the past decade (Wang et al., 2020). Therefore, gender was taken as a control variable in the further analyses. Age was also taken as control variable because of the significant age differences of social anxiety and depression symptoms reported by previous studies (Wang et al., 2020; Guo, 2021).

## Methods

### Participants

The final sample consisted of 185 (92 female) undergraduates who participated in this two-wave survey. Participants were students who attended an introductory psychology class at a Chinese University in North China, aged 18–23 (M = 20.86, SD = 0.97), the majority (98.4%) were Han Chinese. Participants were from various majors (i.e., engineering, education, English). All of them lived in the student dormitories during the study period.

### Instruments

#### Social Achievement Goal Scale

The SAGOS was used to assess social achievement goal orientations among undergraduates from various backgrounds (Horst et al., 2007). A Chinese version (Zhao et al., 2016) with 11 items was used in measuring undergraduates' social mastery and performance (approach and avoid) goals (reference is avoided). Participants were instructed to indicate how much they agree with each of the statements on a scale of 1 (*not at all true of me*) to 5 (*very true of me*). Cronbach's alpha (α) was 0.70 for social mastery goals, 0.70 for social performance-approach goals, and 0.75 for social performance-avoid goals in the current study.

#### Self-Esteem

Self-esteem refers to one's general perceptions of self-worth and satisfaction with self. We used a 9-item Chinese version of self-esteem adapted from Rosenberg's (1965) self-esteem scale (Tian, 2006), with Item 8 (“I wish I could have more respect for myself”) excluded. The exclusion of Item 8 was also supported by another study using a Chinese sample (Sun, 2007). All items were rated on a scale that ranged from 1 (not at all true of me) to 5 (very true of me). Cronbach's alpha was 0.82 in the current study.

#### Social Anxiety Symptoms

We selected the Fear of Social Interaction subscale (FSI) from a Chinese self-report version of the Liebowitz Social Anxiety Scale (He and Zhang, 2004) to indicate undergraduates' social anxious behaviors. The psychometric properties of FSI have been well-established in Chinese samples (He and Zhang, 2004). The 11-items FSI addresses 11 social interactional situations (e.g., meeting strangers), and participants were asked to rate their fear of these situations on a scale from 1 (not at all) to 4 (very much. Cronbach's alpha was 0.79 for the present study.

#### Depressive Symptoms

We borrowed the depression scale from the 21-item version of the Depression Anxiety Stress Scales (Lovibond and Lovibond, 1995). The depression scale, including seven items (e.g., “I felt down-hearted and blue”), was rated on a scale from 1 (*did not apply to me at all*) to 4 (*applied to me most of the time*). Its validity and reliability among student samples were well-established in Chinese settings (Gong et al., 2010). Cronbach's alpha was 0.79 for this study.

### Procedure

In a class session, all class attendants were invited to participate in the study voluntarily; 94% (*N* = 209) agreed to participate in the first wave assessment, 88.5% (*N* = 185) of these participants agreed to participate in the second wave assessment. On each occasion, participants who agreed to join in the study completed a questionnaire packet with a written consent letter. Social achievement goals, self-esteem, social anxiety symptoms, and depressive symptoms were measured in the first wave; 10 months later, social anxiety and depressive symptoms were measured again. The subjects were informed of the study's voluntary nature and could stop at any point if they wanted to. The participants received around 2 dollars incentive for each wave of data collection. The Medical and Scientific Research Ethics Committee of Henan University approved the study.

### Data Analyses

Before conducting further analyses, we first compared the differences between the lost participants and the retained participants in each critical variable. Participants who were missed in the second wave assessment (*N* = 24) did not show significant differences with those participants who completed the two-wave assessment in social achievement goals (*M*_*missing*_ = 4.46, *SD*_*missing*_ = 0.54, *p* > 0.05), social performance-approach goals (*M*_*missing*_ = 2.79, *SD*_*missing*_ = 0.75, *p* > 0.05), social performance-avoid goals (*M*_*missing*_ = 3.13, *SD*_*missing*_ = 0.83, *p* > 0.05), self-esteem (*M*_*missing*_ = 3.00, *SD*_*missing*_ = 0.45, *p* > 0.05), and the symptoms of social anxiety (*M*_*missing*_ = 1.65, *SD*_*missing*_ = 0.38, *p* > 0.05) and depression (*M*_*missing*_ = 1.72, *SD*_*missing*_ = 0.41, *p* > 0.05), but the participants who were missed in the second wave assessment were younger (*M*_*missing*_ = 20.41, *SD*_*missing*_ = 1.10, *p* < 0.05). To address the associations among the targeted variables and estimate whether the links between social achievement goals and the concurrent and subsequent symptoms of social anxiety and depression were mediated by self-esteem, the author conducted path analyses using Amos 21. Three commonly used fit indices were selected to determine the fit of models (Hu and Bentler, 1999): the comparative fit index (CFI, best if > 0.95), the root mean square error of approximation (RMSEA, best if < 0.06), and the standardized root-mean-square residual (SRMR; best if < 0.08).

## Results

### Preliminary Analyses

The data have <1% missing values and are missing at random. We replaced the missing value using expectation maximization (EM) methods in SPSS19.0. Table 1 presents the means, standard deviations, and bivariate correlations of the variables. As expected, males reported higher scores of the longitudinal depressive symptoms, and females reported higher scores of the concurrent and longitudinal social anxiety symptoms. Age was significantly correlated with the longitudinal social anxiety symptoms, implying that older undergraduates reported fewer social anxiety symptoms in the current sample.

**Table 1:** Means, standard deviations, and correlations.

	**M**	**SD**	**1**	**2**	**3**	**4**	**5**	**6**	**7**	**8**	**9**
Gender^ª^	–	–	1								
Age	21.76	1.04	−0.07	1							
Social mastery goals	4.34	0.53	0.08	−0.05	1						
Social performance-approach goals	2.97	0.71	−0.12^†^	0.10	−0.23^**^	1					
Social performance-avoid goals	3.30	0.83	0.00	−0.06	0.27^**^	0.37^**^	1				
Self-esteem	2.92	0.41	−0.05	0.06	0.14^†^	0.24^**^	−0.13^†^	1			
T1 social anxiety symptoms	1.72	0.38	0.22^**^	−0.06	0.09	−0.07	0.28^**^	−0.32^**^	1		
T2 social anxiety symptoms	1.75	0.39	0.19^**^	−0.15^*^	−0.03	−0.11	0.23^**^	−0.29^**^	0.56^**^	1	
T1 depressive symptoms	1.74	0.43	−0.10	−0.05	−0.03	−0.20^**^	0.18^*^	−0.51^**^	0.29^**^	0.29^**^	1
T2 depressive symptoms	1.64	0.42	−0.27^**^	0.11	−0.12^†^	−0.15^*^	0.12^†^	−0.39^**^	0.12	0.16^*^	0.43^**^

ª *Gender 1 = Male, 2 = Female*.

† *p < 0.10*,

* *p < 0.05*,

** *p < 0.01*.

### Path Analyses

**Figure 2:**
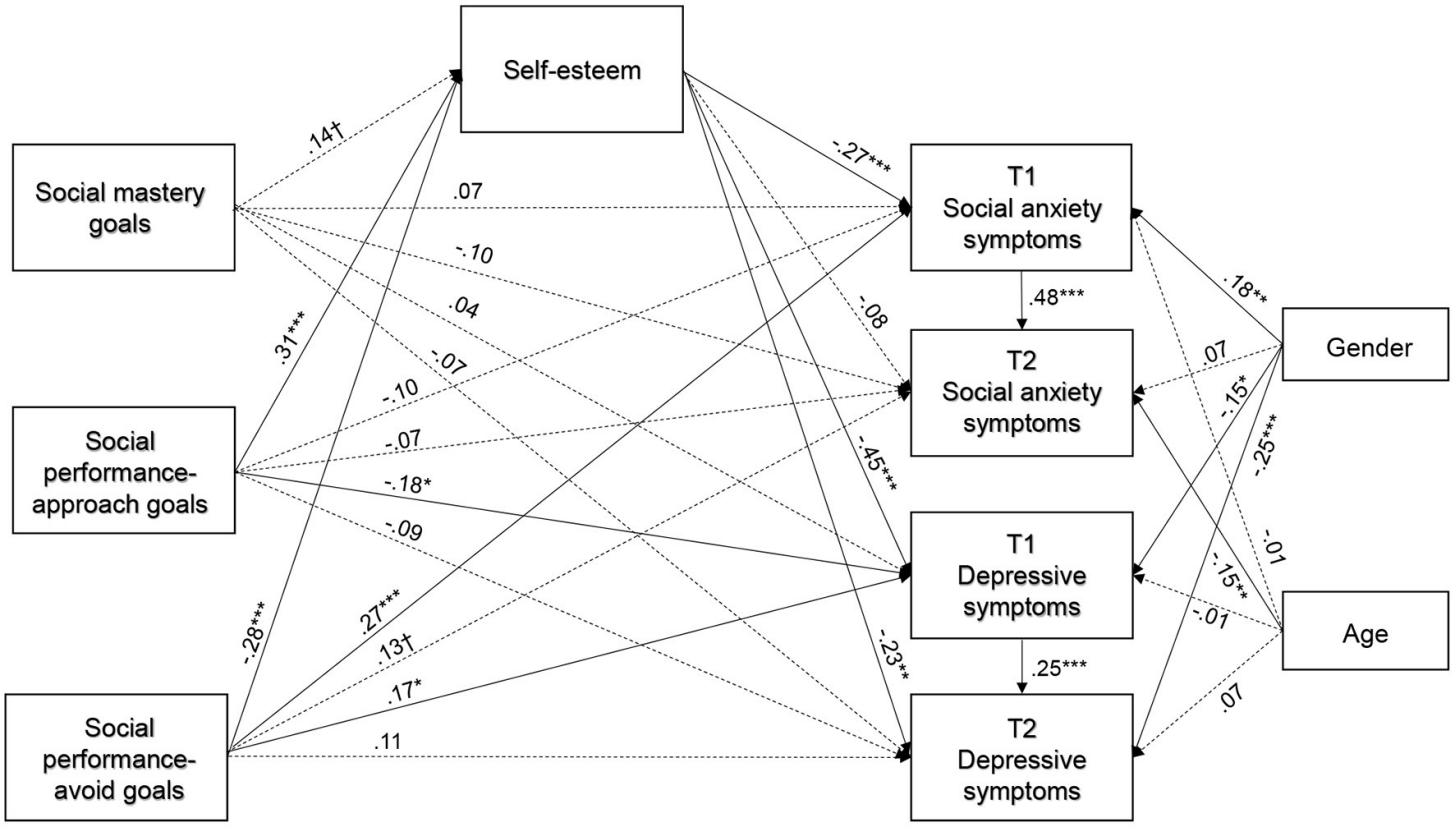
Path analyses model with standardized coefficients.

Path analysis with structural equation modeling was used with the maximum likelihood estimation method. The model includes measures of social achievement goals and self-esteem at Time 1, social anxiety and depressive symptoms at Time 2. We controlled for the effects of gender and age on the social anxiety and depression symptoms tested on two time points. The model showed excellent fit, χ^2^/d*f* ′ = 1.296, *p* = 0.26, CFI = 0.994, RMSEA = 0.040 (0.01–0.11), SRMR = 0.026. As illustrated in the path analyses model (Figure 2), the direct path from social mastery goals to self-esteem was marginally positive (B = 0.14, SE = 0.056, *p* < 0.06); from social performance-approach goals to self-esteem was positive (B = 0.31, SE = 0.043, *p* < 0.01); and from social performance-avoid goals to self-esteem was negative (B = −0.28, SE = 0.037, *p* < 0.01).

As shown in Figure 2, all of the direct paths from social mastery goals to the concurrent social anxiety (B = 0.07, SE = 0.05, *p* > 0.05) and depression symptoms (B = 0.04, SE = 0.05, *p* > 0.05) and to the longitudinal social anxiety (B = −0.10, SE = 0.05, *p* > 0.05) and depression symptoms (B = −0.07, SE = 0.05, *p* > 0.05) were not significant. The link from social performance-approach goals to the concurrent depressive symptoms (B = −0.18, SE = 0.04, *p* < 0.05) was negatively significant, whereas the links from social performance-approach goals to the concurrent (B = −0.10, SE = 0.04, *p* > 0.05) and longitudinal social anxiety symptoms (B = −0.07, SE = 0.04, *p* > 0.05) and the longitudinal depression symptoms (B = −0.09, SE = 0.04, *p* > 0.05) were not significant. The paths from social performance-avoid goals to the concurrent social anxiety (B = 0.27, SE = 0.03, *p* < 0.01) and depression (B = 0.17, SE = 0.04, *p* < 0.05) were positively significant and to the longitudinal symptoms of social anxiety (B = 0.13, SE = 0.03, *p* < 0.07) was marginally positive, but the links from social performance-avoid goals to the longitudinal depression symptoms (B = 0.11, SE = 0.04, *p* > 0.05) were not significant. As expected, the direct paths from self-esteem to the concurrent social anxiety (B = −0.27, SE = 0.06, *p* < 0.01) and depression symptoms (B = −0.45, SE = 0.07, *p* < 0.01) and to the longitudinal depression (B = −0.23, SE = 0.08, *p* < 0.01) were negatively significant; whereas the direct path from self-esteem to the longitudinal social anxiety was not significant (B = −0.08, SE = 0.06, *p* > 0.05).

### Testing Mediation

To test for the proposed indirect effects of social achievement goals on social anxiety and depressive symptoms, a bootstrapping procedure available in Amos 21.0 (2,000 bootstrap samples) was used. According to Shrout and Bolger (2002), an indirect effect is significant at the 0.05 level if the 95% confidence level does not include zero. As shown in Table 2, all of the proposed indirect effects of three types of social achievement goals on the initial and later symptoms of social anxiety and depression *via* self-esteem were significant except for two indirect paths (the paths from social mastery goals through self-esteem to the longitudinal social anxiety symptoms and from social performance-approach goals through self-esteem to the longitudinal social anxiety symptoms). As presented in Table 2, the standardized results indicated that the indirect effects of social mastery goals on the concurrent social anxiety [point estimate = −0.026, SE = 0.017, 95% CI (−0.061, −0.004)] and depression symptoms [point estimate = −0.051, SE = 0.028, 95% CI (−0.102, −0.008)] and on the longitudinal depressive symptoms [point estimate = −0.025, SE = 0.017, 95% CI (−0.063, −0.004)] *via* self-esteem were negatively significant. The chain mediating effects of self-esteem and the initial social anxiety between social mastery goals and the subsequent social anxiety symptoms [point estimate = −0.013, SE = 0.009, 95% CI (−0.032, −0.002)] and the chain mediating effects of self-esteem and the initial depression symptoms between social mastery goals and the later depression symptoms [point estimate = −0.012, SE = 0.008, 95% CI (−0.030, −0.002)] were also significant.

**Table 2:** The indirect effects of self-esteem between social achievement goals and the symptoms of social anxiety and depression.

				**BC 95% CI**	
**Paths**	**Estimates**	**SE**	***p***	**Lower**	**Upper**
**Social mastery goals**					
Social mastery goals → self-esteem → T1 social anxiety symptoms	−0.026	0.017	0.034	−0.061	−0.004
Social mastery goals → self-esteem → T2 social anxiety symptoms	−0.008	0.009	0.102	−0.036	0.001
Social mastery goals → self-esteem → T1 social anxiety symptoms → T2 social anxiety symptoms	−0.013	0.009	0.037	−0.032	0.002
Social mastery goals → self-esteem → T1 depressive symptoms	−0.051	0.028	0.043	−0.102	−0.008
Social mastery goals → self-esteem → T2 depressive symptoms	−0.025	0.017	0.029	−0.063	−0.004
Social mastery goals → self-esteem → T1 depressive symptoms → T2 depressive symptoms	−0.012	0.008	0.029	−0.030	−0.002
**Social performance-approach goals**					
Social performance-approach goals → self-esteem → T1 social anxiety symptoms	−0.044	0.018	0.002	−0.079	−0.021
Social performance-approach goals → self-esteem → T2 social anxiety symptoms	−0.014	0.012	0.116	−0.042	0.001
Social performance-approach goals → self-esteem → T1 social anxiety symptoms → T2 social anxiety symptoms	−0.022	0.009	0.002	−0.039	−0.010
Social performance-approach goals → self-esteem → T1 depressive symptoms	−0.086	0.027	0.001	−0.135	−0.046
Social performance-approach goals → self-esteem → T2 depressive symptoms	−0.042	0.020	0.007	−0.082	−0.015
Social performance-approach goals → self-esteem → T1 depressive symptoms → T2 depressive symptoms	−0.021	0.009	0.001	−0.039	−0.009
**Social performance-avoid goals**					
Social performance-avoid goals → self-esteem → T1 social anxiety symptoms	0.034	0.015	0.002	0.014	0.064
Social performance-avoid goals → self-esteem → T2 social anxiety symptoms	0.011	0.009	0.094	0.001	0.033
Social performance-avoid goals → self-esteem → T1 social anxiety symptoms → T2 social anxiety symptoms	0.016	0.008	0.001	0.007	0.034
Social performance-avoid goals → self-esteem → T1 depressive symptoms	0.065	0.024	0.001	0.031	0.109
Social performance-avoid goals → self-esteem → T2 depressive symptoms	0.032	0.017	0.008	0.010	0.067
Social performance-avoid goals → self-esteem → T1 depressive symptoms → T2 depressive symptoms	0.016	0.007	0.001	0.007	0.032

The indirect effects of social performance-approach goals on the concurrent social anxiety [point estimate =-0.044, SE = 0.018, 95% CI (−0.079, −0.021)] and depression symptoms [point estimate = −0.086, SE = 0.027, 95% CI (−0.135, −0.046)] and the longitudinal depressive symptoms [point estimate = −0.042, SE = 0.020, 95% CI (−0.082, −0.015)] *via* self-esteem were significant. The previous symptoms of social anxiety and depression significantly predicted the subsequent social anxiety (B = 0.48, SE = 0.07, *p* < 0.01) and depression symptoms (B = 0.25, SE = 0.07, *p* < 0.01). The chain mediating effects of self-esteem and the initial social anxiety between social performance-approach goals and the subsequent social anxiety symptoms [point estimate = −0.022, SE = 0.009, 95% CI (−0.039, −0.010)] and the chain mediating effects of self-esteem and the initial depression symptoms between social performance-approach goals and the later depression symptoms [point estimate = −0.021, SE = 0.009, 95% CI (−0.039, −0.009)] were significant. As for social performance-avoid goals, the indirect effects of social performance-avoid goals on the concurrent social anxiety [point estimate = 0.034, SE = 0.015, 95% CI (0.014, 0.064)] and depression symptoms [point estimate = −0.065, SE = 0.024, 95% CI (0.031, 0.109)] and the longitudinal social anxiety [point estimate = 0.011, SE = 0.009, 95% CI (0.001, 0.033)] and depression symptoms [point estimate = 0.032, SE = 0.017, 95% CI (0.010, 0.067)] *via* self-esteem were all significant. The chain mediating effects of self-esteem and the initial social anxiety between social performance-avoid goals and the subsequent social anxiety symptoms [point estimate = 0.016, SE = 0.008, 95% CI (0.007, 0.034)] and the chain mediating effects of self-esteem and the initial depression symptoms between social performance-avoid goals and the subsequent depression symptoms [point estimate = 0.016, SE = 0.007, 95% CI (0.007, 0.032)] were significant.

## Discussion

The current study evaluated whether social achievement goals, self-esteem, and social anxiety and depression symptoms measured at an initial assessment significantly predicted the presence of social anxiety and depression symptoms measured 10 months later. The study also explored whether the relationships between social achievement goals and the concurrent and longitudinal social anxiety and depression symptoms were mediated by self-esteem and whether the chain effects of self-esteem and the initial levels of social anxiety and depression symptoms existed between the links from social achievement goals to the subsequent psychopathology symptoms. As presented in previous studies (Shim and Ryan, 2012; Shim et al., 2012, 2017; Hiller et al., 2017; Gao et al., 2022), meaningful associations were found between social achievement goals, self-esteem, and concurrent and longitudinal social anxiety and depression symptoms, which provided insight into potential risk factors and resources for undergraduates' affective wellbeing. In particular, self-esteem was found to mediate the majority of associations between social achievement goals and the concurrent and longitudinal social anxiety and depression symptoms. The chain mediation effects of self-esteem and the concurrent symptoms of social anxiety and depression demonstrate the possible indirect effects of social achievement goals on the development of social anxiety and depression through self-esteem and initial social anxiety and depression symptoms.

Contrary to Shim et al.'s (2017) study, path analyses showed that the endorsement of social performance-approach goals was associated with the decrease of students' concurrent depressive symptoms. The process of trying to prove social competence and winning reputation in social situations appears to provide some protective effects on soothing concurrent depressive symptoms. However, this finding supports Kuroda and Sakurai's (2003) results using Korean samples. These inconsistent findings suggest further investigations of the conditions under which social performance-approach goals contribute to affective outcomes. Findings also suggest a positive association between social performance-avoid goals and the concurrent and longitudinal social anxiety and depressive symptoms, which is consistent with previous findings using undergraduate samples (Kuroda and Sakurai, 2011; Shim and Ryan, 2012; Shim et al., 2017), suggesting that this goal orientation increases undergraduates' social anxious behaviors and depressive symptoms. Inconsistent with these studies, no significant associations have been found between social performance-approach goals and the concurrent social anxiety symptoms and between social mastery goals and the concurrent social anxiety and depression symptoms. It is worth noting that all three types of social achievement goals were not direct predictors of the longitudinal symptoms of social anxiety and depression. One possible reason might be that the symptoms of depression and anxiety are context-sensible, which may be affected strongly by recent events (Samios et al., 2019; Casline et al., 2020). From another perspective, these results suggest the existence of mediation mechanisms, such as self-esteem, functioning between these links.

All three types of social achievement goals are potential predictors of self-esteem. As proposed previously (Shim et al., 2012; Kim and Park, 2021), social mastery goals were positively associated with self-esteem at a marginal level, whereas social performance-avoid goals were negatively associated with self-esteem. These findings suggest that pursuing social mastery goals in developing social relationships boosts undergraduates' self-worth, but the strivings to hide inadequacy and avoid negative evaluation in social contexts destroy one's self-worth. Inconsistent with Shim and Ryan (2012), a positive association between social performance-approach goals and self-esteem has been found, indicating that the pursuit of proving one's social competence and winning others' positive evaluation boosts undergraduates' self-worth. One explanation might be that the association between social performance-approach goals and self-esteem is moderated by Chinese culture, which values the significance of personal connections (Hsiung, 2013). Being successful at some socially valued activity is “a major source of self-esteem” (Covington, 1984, p. 8). Another explanation might be that people endorsing more social performance-approach goals value and demonstrate the number of their friends. The increased quantity of social friends then strengthens one's self-esteem (Denissen et al., 2008; Stinson et al., 2008).

As the theories (Beck, 1967; Clark and Wells, 1995) and empirical studies (Cheng et al., 2015; Li et al., 2015; Abdollahi and Abu Talib, 2016; Rieger et al., 2016; Hiller et al., 2017; Gao et al., 2022) suggested, low self-esteem increases the risks of experiencing social anxiety and depression symptoms in the current study. The findings support the proposed mediation effects of self-esteem on the links from three types of social achievement goals to the concurrent and longitudinal social anxiety and depression symptoms except on the links from social mastery goals and social performance-approach goals to the longitudinal social anxiety symptoms. Specifically, the associations between social mastery goals and the concurrent social anxiety and between social mastery goals and the concurrent and longitudinal depression symptoms were partly explained by self-esteem. It suggests that the pursuit of social mastery goals (developing a high-quality relationship and personal growth) in the process of social relationship strengthens one's social competence and positive self-evaluation (Horst et al., 2007; Hapsari and Sholichah, 2021), which, in turn, decreases the risks of suffering social anxiety and depression symptoms concurrently or longitudinally, or both. Furthermore, results suggest that endorsing social performance-approach goals (trying to prove oneself and winning a positive reputation) protects people from social anxiety and depressive symptoms through enhancing undergraduates' self-esteem. Finally, as proposed, endorsing an avoidance approach in building and maintaining social relationships weakens one's evaluation of social self, which, in turn, increases the risks of experiencing concurrent and longitudinal social anxiety and depressive symptoms.

The initial tested social anxiety and depression symptoms have consistently predicted the subsequent social anxiety and depression symptoms, especially for the social anxiety symptoms. This might be the reason why social achievement goals and self-esteem could not predict the subsequently tested social anxiety symptoms directly or indirectly. In other words, students identified with higher social anxiety and depression symptoms demonstrate a higher risk for subsequent social anxiety and depression symptoms (Rodebaugh et al., 2015; Santini et al., 2020). The chain mediation analyses revealed that social achievement goals might influence students' longitudinal social anxiety and depressive symptoms through the chain mediation of self-esteem and the initial levels of the social anxiety and depressive symptoms. These findings suggest that social achievement goals may first affect individuals' social anxiety and depressive symptoms *via* self-esteem, and then the initial states of the social anxiety and depression symptoms predict the follow-up tested symptoms of social anxiety and depression.

### Applications

Social achievement goal theory describes the different achievement goals that individuals endorse in developing friendship, and the current study evidences the crucial roles of these goals in the development of undergraduates' social anxiety and depression. Several implications may follow from this study. For example, it would be helpful to introduce the social achievement goal theory into the relationship education courses in university. Teachers can explain the meaning and possible consequences of different goals in developing new friendships and help students establish appropriate social achievement goals, thereby improving students' friendship quality and diminishing the negative impact of inappropriate goals on individuals' self-development and emotional health. Meanwhile, college students' perceived interpersonal stress and relationship quality have important applications on their social development and wellbeing (Rodebaugh et al., 2015; Spithoven et al., 2017). The psychological counseling practice in higher education could benefit from social achievement goal theory research. Specifically, practitioners of university counseling centers could borrow the social achievement goal theory to help students analyze the difficulties and pressures they face in interpersonal relationships, from why students want to develop a relationship and what they want to achieve in their friendship. Intervention programs targeting the psychological development of students may try to increase students' social mastery-approach goals and decrease their social performance-avoid goals. These attempts may improve students' self-esteem and social competence and decrease their vulnerability to social anxiety and depression.

### Limitations and Future Directions

Several limitations should be considered in interpreting these findings. First, the sample size of this study was small, which potentially limited the power of the current results. Further studies with a large sample size are needed to replicate and strengthen the current research findings. Second, some social anxiety symptoms translate into social anxiety disorder over time, but experiencing social anxiety symptoms is not equal to being identified as having a clinical social anxiety disorder. The same situation applies to depression symptoms and the major depression disorder. The protective factors suggested in this study for decreasing social anxiety and depression symptoms may not be effective for tampering with the psychiatric disorder of social anxiety and depression. Future studies could consider using a clinical sample to reexamine the proposed model of this study. Third, this study did not control the effects of comorbid psychiatric disorders such as comorbid post-traumatic disorder or generalized anxiety disorder, which may bias the current findings. Finally, although we proposed that social achievement goals may lead to self-esteem, the change of self-esteem might lead young people to readjust their goal pursuits in building and maintaining their relationships. Future studies testing variables at more time points and controlling for baseline mental health can provide better evidence for the relationship between social achievement goals, self-esteem, and other mental health indicators.

### Conclusions

In closing, the present study further supports that social achievement goals in developing relationships are meaningful predictors of undergraduates' social anxiety and depression symptoms. The function of self-esteem as the underlying mechanism in explaining majority associations between social achievement goals and the concurrent and longitudinal symptoms of social anxiety and depression deserves further investigation. The concern of why people want to develop social relationships can further understand the development of the psychopathology of social anxiety and depression.

## Data Availability Statement

The raw data supporting the conclusions of this article will be made available by the authors, without undue reservation.

## Ethics Statement

The studies involving human participants were reviewed and approved by Committee on Biomedical Research Ethics, Henan University. The patients/participants provided their written informed consent to participate in this study.

## Author Contributions

The author confirms being the sole contributor of this work and has approved it for publication.

## Funding

The study was funded by the Youth Foundation of Humanity and Social Sciences from the Ministry of Education of China, Grant Number 18YJC190034 and the Henan Province Scientific and Technique Foundation, Grant Number 212102310094.

## Conflict of Interest

The author declares that the research was conducted in the absence of any commercial or financial relationships that could be construed as a potential conflict of interest.

## Publisher's Note

All claims expressed in this article are solely those of the authors and do not necessarily represent those of their affiliated organizations, or those of the publisher, the editors and the reviewers. Any product that may be evaluated in this article, or claim that may be made by its manufacturer, is not guaranteed or endorsed by the publisher.
